# Whole Exome Sequencing Leading to the Diagnosis of Dysferlinopathy with a Novel Missense Mutation (c.959G>C)

**DOI:** 10.1155/2016/9280812

**Published:** 2016-04-19

**Authors:** Abhisek Swaika, Nicole J. Boczek, Neha Sood, Kimberly Guthrie, Eric W. Klee, Ankit Agrawal, Elliot L. Dimberg, Sikander Ailawadhi

**Affiliations:** ^1^Division of Hematology-Oncology, Mayo Clinic, Jacksonville, FL 32224, USA; ^2^Center for Individualized Medicine, Mayo Clinic, Rochester, MN 55905, USA; ^3^Center for Individualized Medicine, Mayo Clinic, Jacksonville, FL 32224, USA; ^4^Department of Neurology, Mayo Clinic, Jacksonville, FL 32224, USA

## Abstract

Dysferlinopathy is an uncommon, progressive muscular dystrophy that has a wide phenotypic variability and primarily supportive management (Nguyen et al., 2007; Narayanaswami et al., 2014). Amyloid myopathy is a distinct, rare disorder that can present similarly to inflammatory myopathies and requires a high clinical suspicion for early intervention to prolong survival. Amyloid myopathy is typically associated with other systemic manifestations of amyloidosis, but rare cases of isolated amyloid myopathy have been described (Mandl et al., 2000; Hull et al., 2001). Positive Congo red stains on tissue biopsy remain the gold standard for diagnosis (Spuler et al., 1998; Karacostas et al., 2005). A high clinical suspicion and meticulous diagnostic workup that includes novel techniques are necessary for identifying these rare disorders. We report a middle-aged man with progressive leg muscle weakness who was initially treated as having amyloid myopathy but was later diagnosed as having dysferlinopathy by Whole Exome Sequencing (WES) analysis. We also report a novel missense mutation (c.959G>C) to help correlate in any patient with presumed dysferlinopathy and to add to the already known genotype of this disorder.

## 1. Clinical Report

**Figure 1:**
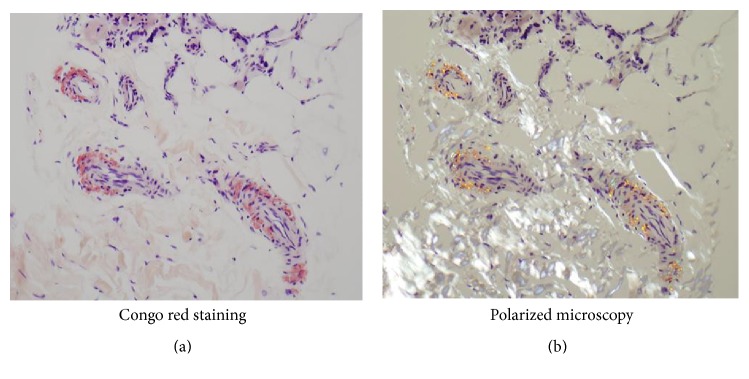
Perivascular and endomysial congophilic deposits on (a) H&E and (b) polarized microscopy.

A 43-year-old Caucasian man with progressive proximal bilateral leg muscle weakness (over the span of 1 year) and increased creatine kinase (1537 u/L) and aldolase levels (23.7 u/L) was treated with etanercept and methotrexate for presumed inflammatory myopathy under the care of a rheumatologist. He had a 20-year history of stable right calf atrophy, which he had attributed to a prior football injury, but he reported no other neuromuscular symptoms. He also did not report any other constitutional symptoms. A neurological consult and evaluation that included electromyography and nerve conduction testing revealed nonspecific myopathy. After six months of treatment without significant improvement, he underwent a vastus lateralis muscle biopsy that revealed perivascular and endomysial congophilic deposits (Figure 1). A repeat biopsy confirmed these findings, but there were no associated dystrophic changes, which led to the diagnosis of amyloid myopathy. He was referred to our institution where an extensive evaluation for systemic amyloidosis, including bone marrow biopsy, biochemical testing, and imaging, was negative. Fat aspirate and rectal biopsy were also inconclusive. Familial history was noncontributory for any similarly affected individuals. Mutations for transthyretin and fibrinogen-alpha-gene- (FGA-) related familial visceral amyloidosis were negative. Repeat electromyography and nerve conduction testing again revealed nonspecific myopathy. The muscle biopsy slides were reexamined with no additional findings. Considering the worsening of his weakness as well as new progressive involvement of bilateral proximal arm muscles, treatment was initiated with chemotherapy, including cyclophosphamide, bortezomib, and dexamethasone (CyBorD) based on a diagnosis of isolated amyloid myopathy [1]. He tolerated the treatment well but did not have any clinical improvement after three completed cycles; the diagnosis was again questioned. Following a medical genetics consult, Whole Exome Sequencing (WES) was performed for the patient using a commercially available test. The test reports that 90% of bases are expected to have a quality score of Q20 or higher, and their exome analysis augments coverage in over 7,500 additional genes currently associated with human disease. In addition, all reported genes in the Personalis WES report are covered >99.9% with a depth of at least 20x.

**Figure 2:**
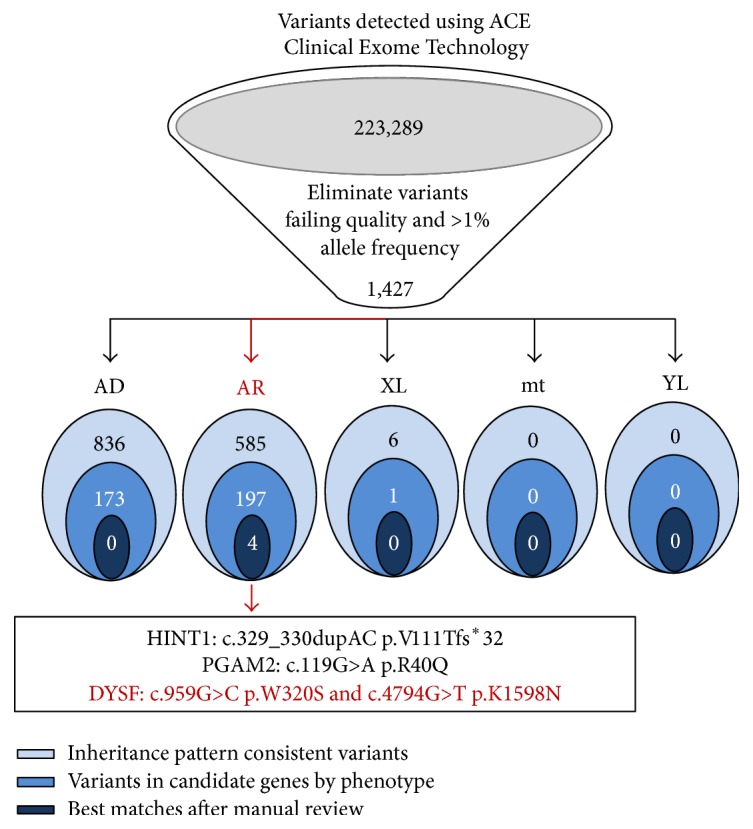
Whole Exome Sequencing analysis. Whole Exome Sequencing was completed at Personalis and variants were detected using their ACE Clinical Exome Pipeline. Variants were filtered by Personalis as described above, first with the elimination of variants with poor quality and >1% frequency. Then, variants were sorted based on inheritance pattern (AD: autosomal dominant, AR: autosomal recessive, XL: X-linked, mt: mitochondria, and YL: Y-linked) and variants were kept if the gene matched the phenotype. The variants that were the best matches after manual review were reported. Four variants in three genes (HINT1, PGAM2, and DYSF) passed all filtering steps. Two variants were detected in DYSF and information regarding the variants is described in Table 1.

Overall, four variants passed all of the test filtering steps,* HINT1*: c.329_330dupAC p.V111Tfs^*∗*^32;* PGAM2*: c.119G>A p.R40Q;* DYSF*: c.959G>C p.W320S; and c.4794G>T p.K1598N (Figure 2 and Table 1).* HINT1* mutations are associated with neuromyotonia and axonal neuropathy, and mutations in* PGAM2 *are associated with glycogen storage disease X. These disorders are autosomal recessive, and because only one variant was identified, these variants were not likely associated with the phenotype or consistent with the clinical presentation of the patient. Two variants of uncertain significance were identified in the* DYSF* gene encoding dysferlin (Figure 2 and Table 1). Sanger sequencing of the paternal sample established that only the c.959G>C variant was of paternal origin, while c.4794G.T was not present in the father's sample. Testing from his unaffected mother was not possible due to logistic reasons, and he had no other siblings. Therefore, it was possible that the two* DYSF* variants were in trans, one inherited from each parent, or the second* DYSF* variant, c.4794G>T, was de novo, in which the variants could be in cis or trans.* DYSF* is known to be associated with Miyoshi myopathy, limb-girdle muscular dystrophy 2B (LGMD2B), and distal myopathy with anterior tibial onset, all of which are autosomal recessive conditions. The c.959G>C variant is novel, whereas c.4794G>T has been previously described in an individual with limb-girdle muscular dystrophy (LGMD) type 2B in the Leiden Muscular Dystrophy (DYSF) pages (http://www.dmd.nl/nmdb/home.php?select_db=DYSF). This led to further immunohistochemical testing on the previously obtained muscle biopsy specimen from our patient that demonstrated an absence of dysferlin staining, thus establishing the diagnosis of dysferlinopathy (LGMD2B).

## 2. Discussion

Dysferlinopathies have wide phenotypic variability, even within families, including limb-girdle muscular dystrophy, Miyoshi myopathy, distal anterior compartment myopathy, pseudometabolic myopathy, and asymptomatic hyperCKemia [2]. Dysferlinopathy is characterized by the loss (or significant reduction to <10% normal levels) of the dysferlin protein (encoded by the* DYSF* gene) in the skeletal muscle of affected patients and has an autosomal recessive mode of inheritance [3]. The typical age of onset ranges from 15 to 35 years. These patients often have very high serum CK levels (1000–40,000 units/L or more) and early weakness of proximal leg muscles [4]. Arm weakness may occur with progression, but facial and bulbar muscles are generally not affected. Cardiac or pulmonary involvement is also uncommon.

LGMD2B is usually slowly progressive and can cause wheelchair dependence within 10 to 20 years after onset [5]. Prior to onset, the patient is usually normal, and some individuals even excel at athletics. The management of LGMD is supportive; no disease-modifying treatments are available [6].

A high clinical suspicion is necessary to diagnose this rare disease, which often leads to delay in diagnosis. Progressive muscle weakness and muscle atrophy mainly involving the proximal limb muscles, particularly if there is a family history of a similar disorder, should raise the suspicion of a muscular dystrophy. Due to the considerable overlap and heterogeneity among the various subtypes, these patients are best served by a referral to a neuromuscular disorder specialist. Targeted genetic and protein testing should mostly precede muscle biopsies in these disorders [6]. WES has also recently been reported to provide a successful LGMD diagnosis in 45% of 60 families within a difficult-to-diagnose cohort of LGMD patients, and it has expanded the clinical phenotypes associated with known myopathy genes [7].

Amyloid deposits may be seen in the blood vessel walls, perimysial connective tissue, or endomysium surrounding muscle fibers in muscle biopsies of dysferlin-deficient patients [8]. Amyloid is derived from the mutant dysferlin and most likely represents a fragment of the full-length protein that has been degraded and then folded to form a *β*-pleated sheet with typical green birefringence under polarized light [8].

Isolated presentation of amyloid myopathy is also a very rare entity, but it can be diagnosed as the initial presenting feature of systemic amyloidosis [9, 10]. Gertz and Kyle only reported 12 cases of amyloid myopathy in a large retrospective review of 1596 patients with systemic amyloidosis [11]. The presenting symptoms can be nonspecific, but most reported cases presented with proximal muscle weakness [11, 12]. It can also mimic inflammatory myopathies [13, 14]. Congo red stains on the muscle biopsy after unusual findings on magnetic resonance imaging (MRI) or positron emission tomography (PET)/computed tomography (CT) scanning could aid in diagnosis [15, 16].

A high clinical suspicion is necessary to diagnose these rare disorders; our case highlights the importance of recognizing clinical clues and integration of available diagnostic tools, including WES, to achieve this goal and prevent any unnecessary harmful interventions. We also report a novel missense mutation (c.959G>C) to help correlate in any patient with presumed dysferlinopathy and to add to the already known genotype of this disorder.

## Figures and Tables

**Table 1:** 

DYSF	c.959G>C p.W320S, paternal	c.4794G>T p.K1598N
Interpretation	VUS	VUS

ESP & ExAC	ESP: NR; ExAC: NR	ESP: 2/8598; ExAC: 4/121348

SIFT/PolyPhen/MutationTaster	Damaging/probably damaging/disease causing	Tolerated/probably damaging/disease causing

Location (gene; protein)	Exon 11; ferlin family domain	Exon 43; C2 domain F

Previously reported variants in affected individuals	This variant has never been reported; however, there is a previously reported variant in an affected individual in close proximity: p.L322P	This variant has been reported in one affected individual and there are two variants in affected individuals in close proximity: c.4794+1G>A and p.Gly1547_Lys1598del

Diseases associated with DYSF variants	Autosomal recessive Miyoshi muscular dystrophy 1, autosomal recessive limb-girdle muscular dystrophy type 2B, autosomal recessive distal myopathy with anterior tibial onset	

VUS: variant of uncertain significance; ESP: National Heart Lung and Blood Institute Exome Sequencing Project; ExAC: Exome Aggregation Consortium; NR: not reported.
